# Supplementary material for: Transmission and dynamics of mother-infant gut viruses during pregnancy and early life
Source: Nat Commun. 2024 Mar 2;15:1945. doi: 10.1038/s41467-024-45257-4 (PMC10908809; doi:10.1038/s41467-024-45257-4)
Supplement: Supplementary file 1 — Supplementary Information [file 41467_2024_45257_MOESM1_ESM.pdf]

Supplementary information: Transmission and Dynamics of Mother-Infant Gut Viruses during Pregnancy and Early Life

S. Garmaeva and T. Sinha et al.

Supplementary Figure 1: Study population, vOTU richness in VLP metaviromes, and bacteriome stability.

Supplementary Figure 2: Percentage of sample composition classified into biome fractions and the abundance of these fractions.

Supplementary Figure 3: Correlation between infant vOTU-host-genus aggregates and bacterial genera and their dynamics in infants.

Supplementary Figure 4: Temperate phages in MGS metaviromes, gut virome associations with phenotypes and within-individual virus strain variation.

Supplementary Figure 5: Within-individual bacterial strain variation

Supplementary Figure 6: Example of virus-host co-transmission not rooted in the maternal gut: a temperate phage of *Bifidobacterium scardovii*

Supplementary Figures

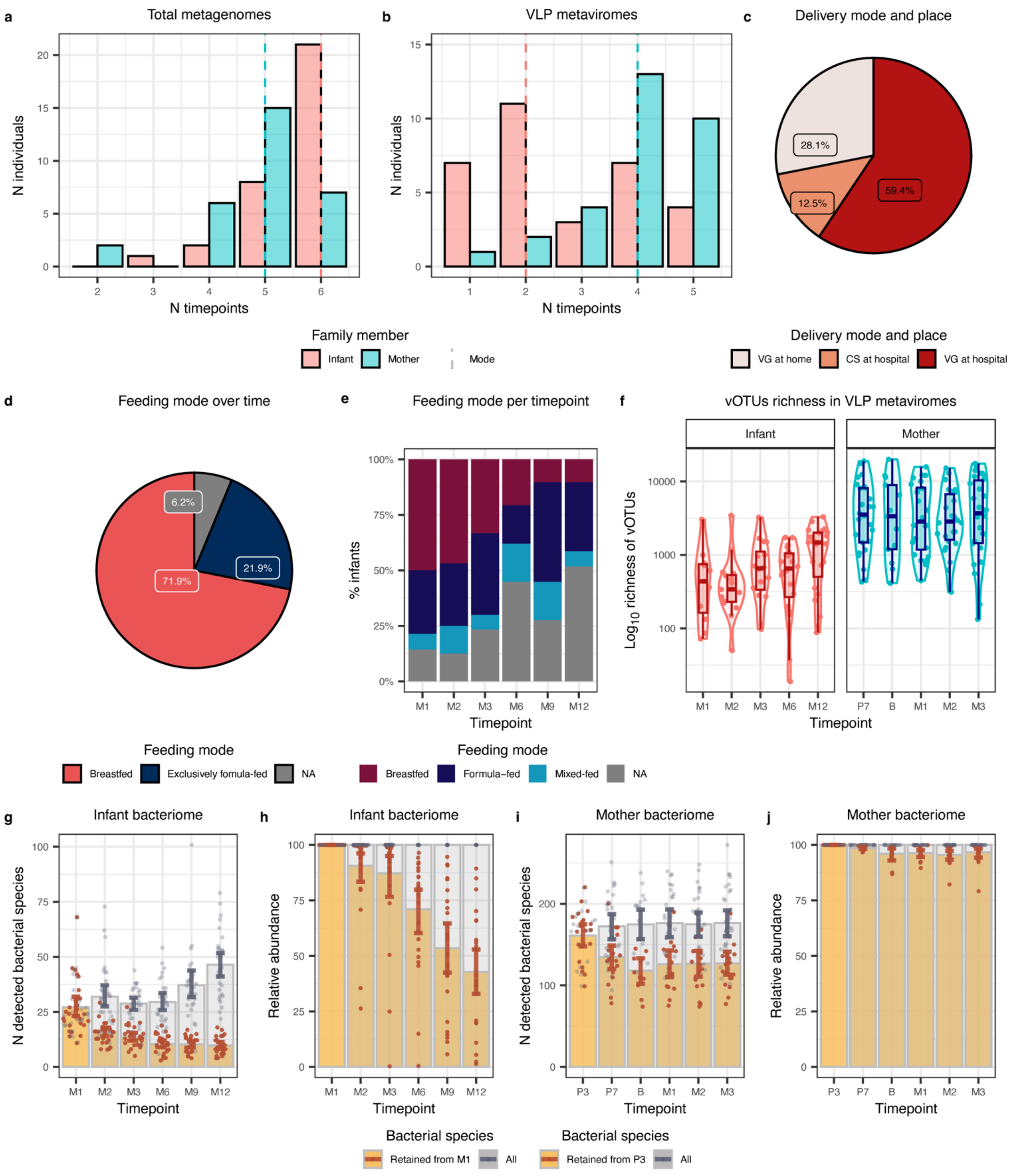

Supplementary Figure 1: Study population, vOTU richness in VLP metaviromes, and bacteriome stability.

a, **b**, Distribution of the number of available timepoints in **a**, total metagenomes and **b**, VLP metaviromes for infants (pink) and mothers (cyan). Dashed lines of respective colours depict the mode per family member. **c**, Distribution of infants by delivery mode and location of delivery. Colours of the pie chart depict the mode and location of delivery: vaginal delivery (VG) at home (beige), cesarean section (CS) at hospital (orange), VG at hospital (red). **d**, Distribution of the infant feeding mode across all timepoints. **e**, Per-timepoint breakdown of the infant feeding mode. Y-axis of the stacked bar plot depicts the percentage of infants within each feeding mode category at the given timepoint. In **d**, **e**, grey denotes unavailable feeding practices data (NA). **f,** vOTU richness in infant and maternal VLP metaviromes over time (Supplementary Data 2). The sample size for the analysis using VLP metaviromes varies by timepoint and family member and is depicted in Fig. 1a. Each boxplot visualizes the median, hinges (25th and 75th percentiles), and whiskers extending up to 1.5 times the interquartile range from the hinges. **g**, Number and **h**, relative abundance of bacterial species retained from month 1 in infants at months 2, 3, 6, 9 and 12 after birth. n=28 infants with available total metagenome sequencing at M1 were examined over 6 timepoints. n(M1)=28, n(M2)=28, n(M3)=26, n(M6)=25, n(M9)=25, n(M12)=25. **i**, Number and **j,** relative abundance of bacterial species retained from the 3rd month of pregnancy in mothers to the 7th month of pregnancy, delivery, and months 1, 2, 3 after delivery. n=20 mothers with available total metagenome sequencing at P3 were examined over 6 timepoints. n(P3)=20, n(P7)=17, n(B)=11, n(M1)=15, n(M2)=17, n(M3)=19. In **g-j**, bar colours show the retained (yellow) and all (grey) bacterial species per sample. The 95% confidence intervals (CI) for the means are depicted in brown and grey whiskers for retained and all bacterial species, respectively. Source data are provided as a Source Data file.

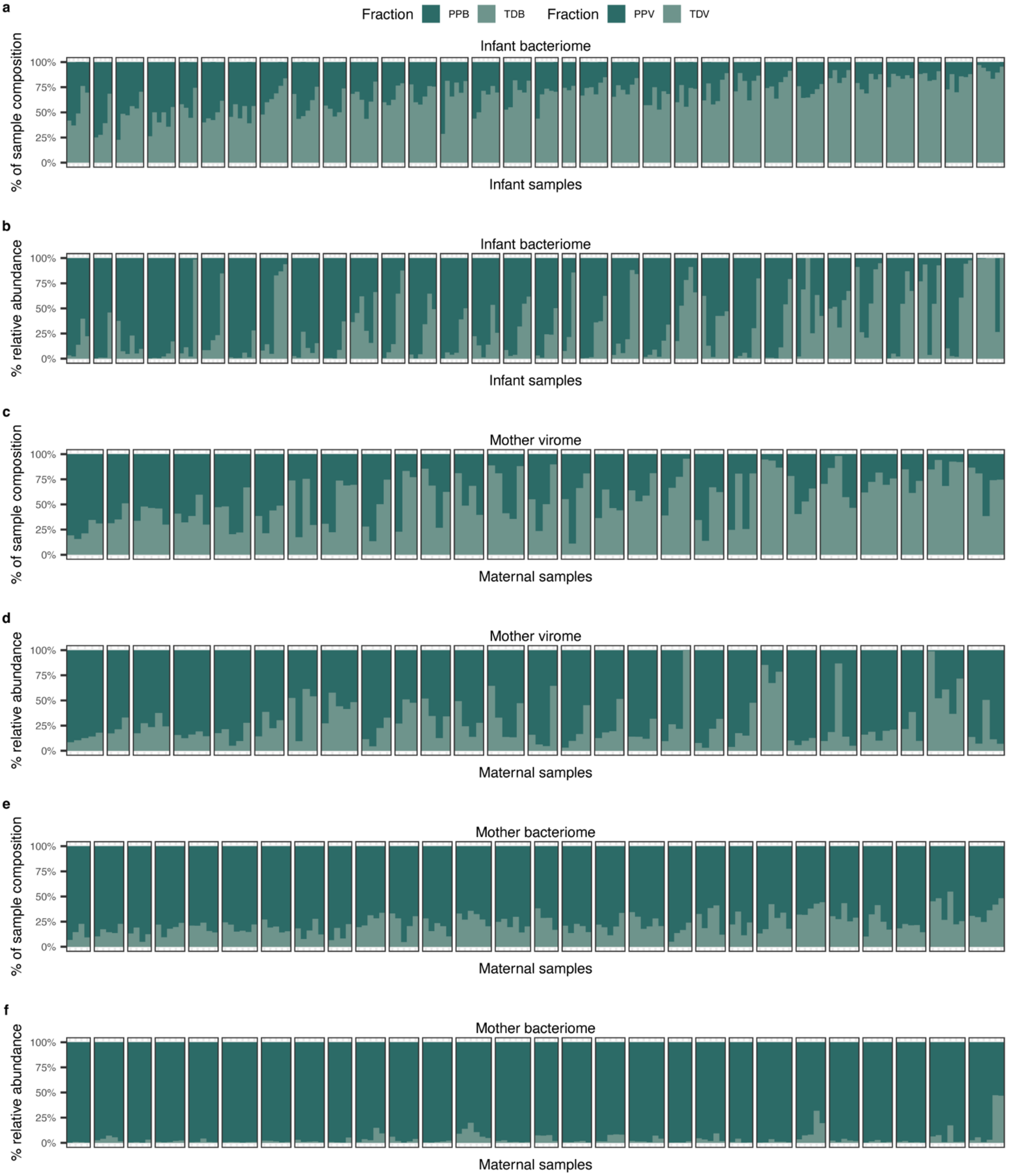

Supplementary Figure 2: Percentage of sample composition classified into biome fractions and the abundance of these fractions.

a, Percentage of infant bacteriome classified into personal persistent bacteriome (PPB) and transiently detected bacteriome (TDB) fractions. PPB and TDB are defined

based on the species persistence in the bacteriome of 32 infants. PPB (deep teal) includes bacterial species present in >= 75% of samples from an individual, while TDB (muted seafoam) includes those present in less than 75% of an individual's samples. **b,** Relative abundance of PPB and TDB fractions in the infant bacteriome. **c,** Percentage of maternal VLP metavirome classified into personal persistent virome (PPV) and transiently detected virome (TDV) biome fractions. PPV and TDV are defined based on the vOTUs persistence in the VLP metaviromes of 27 mothers with at least 3 longitudinal samples. PPV (deep teal) includes vOTUs present in >= 75% of samples from an individual, while TDVs (muted seafoam) includes those present in less than 75% of the samples of an individual. **d,** The relative abundance of PPV and TDV in maternal virome. **e,** Percentage of maternal bacteriome classified into PPB and TDB fractions in 28 mothers with at least 3 longitudinal total metagenome samples. **f,** The relative abundance of PPB and TDB in maternal bacteriome. In **a**-**f**, each facet depicts one timepoint of a family member (infant or mother), and every outlined group of facets depicts one individual. Source data are provided as a Source Data file.

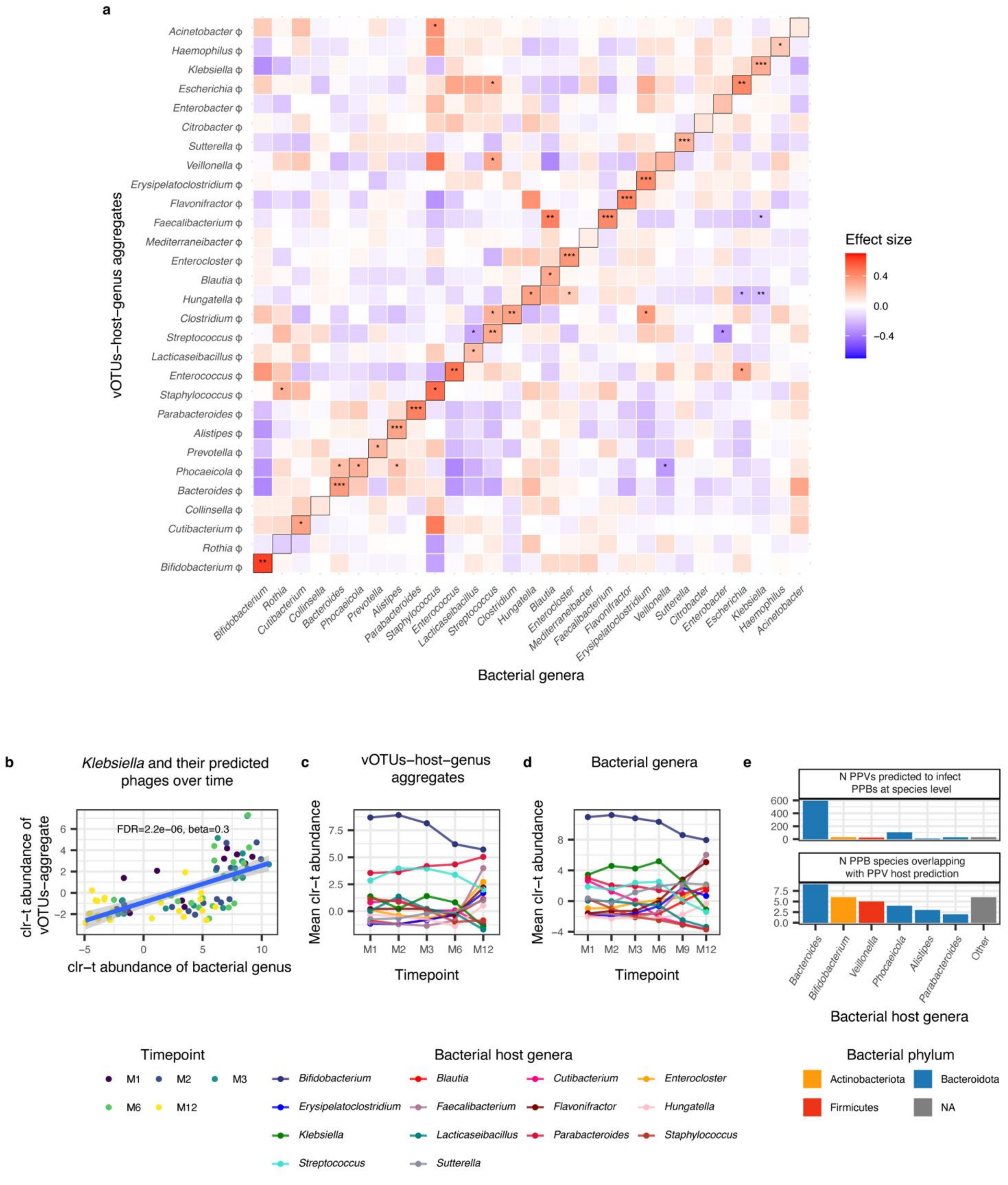

Supplementary Figure 3: Correlation between infant vOTU-host-genus aggregates and bacterial genera and their dynamics in infants.

a, Phage-bacterial host associations in the infant gut: heatmap of linear mixed-effects models (LMM) association results between bacterial genera and vOTUs, aggregated

by predicted bacterial hosts at the genus level, found in at least 20% of infant VLP metaviromes. LMM included all paired vOTU aggregates and bacterial genera present in at least 10% of concurrent longitudinal VLP metaviromes and total metagenomes of the 32 infants. The resulting p-values were corrected using Benjamini–Hochberg method. The colour of the heatmap tile represents the effect size, and the asterisks indicate FDR significance. *** indicates FDR < 0.001, ** FDR < 0.01, and * FDR < 0.05. **b**, Association of *Klebsiella* and its phages over time in the infant gut: a scatterplot showing the correlation between the centred log ratio (clr) transformed vOTU abundances, aggregated based on their predicted host *Klebsiella* (Y-axis) and the clr-transformed abundance of the bacterial genus *Klebsiell*a (X-axis). The dot colour shows the infant timepoint. **c**, Temporal dynamics of vOTU host aggregates in infants. Only FDR-significant (FDR < 0.05) vOTU aggregates prevalent in >20% of infant VLP metaviromes and associated with their predicted host at the genus level and timepoint are shown. **d,** Temporal dynamics of the infant bacterial genera predicted as hosts for vOTU aggregates. LMMs were used to evaluate the relationship between vOTU host aggregates or bacterial genus abundance and timepoint (Supplementary Data 21, 22). **e,** Predicted hosts of personal persistent viruses (PPV): top bar plot shows the count of PPVs predicted to infect personal persistent bacteria (PPB) at the species level. The bottom plot shows the overlap of PPBs and PPVs categorized by the PPVs' host prediction within the prevalent genera. Source data are provided as a Source Data file.

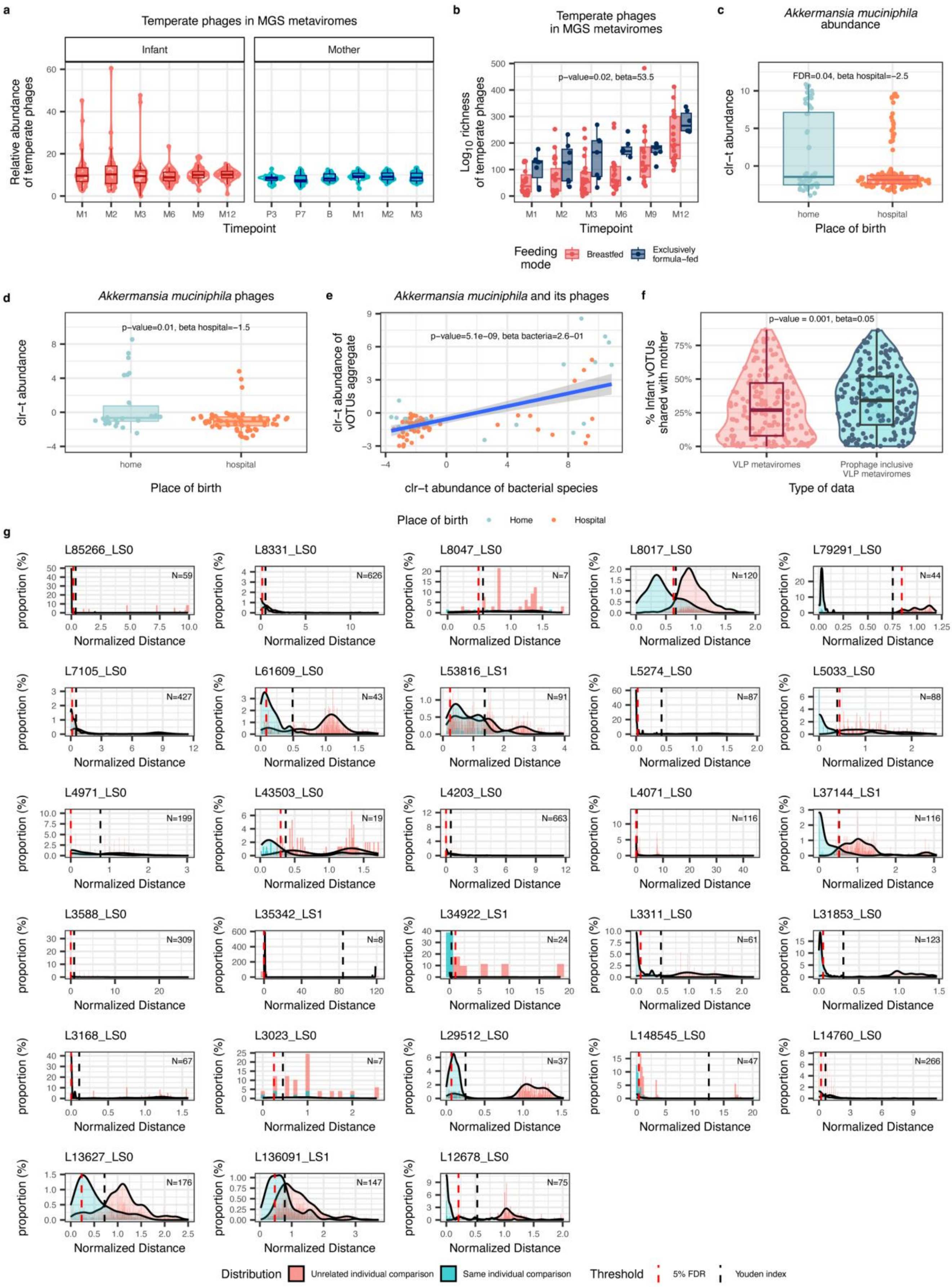
a
Temperate phages in MGS metaviromes
Infant
Mother
Relative abundance of temperate phages
Timepoint
M1 M2 M3 M6 M9 M12
P3 P7 B M1 M2 M3
b
Temperate phages in MGS metaviromes
p-value=0.02, beta=53.5
Log10 richness of temperate phages
Timepoint
Feeding mode
Breastfed
Exclusively formula-fed
c
Akkermansia muciniphila abundance
FDR=0.04, beta hospital=-2.5
clr-t abundance
home
hospital
Place of birth
d
Akkermansia muciniphila phages
p-value=0.01, beta hospital=-1.5
clr-t abundance
Place of birth
e
Akkermansia muciniphila and its phages
p-value=5.1e-09, beta bacteria=2.6-01
clr-t abundance of vOTUs aggregate
clr-t abundance of bacterial species
f
p-value = 0.001, beta=0.05
% Infant vOTUs shared with mother
VLP metaviromes
Prophage inclusive VLP metaviromes
Type of data
Place of birth
Home
Hospital
g
L85266_LS0 N=59
L8331_LS0 N=626
L8047_LS0 N=7
L8017_LS0 N=120
L79291_LS0 N=44
L7105_LS0 N=427
L61609_LS0 N=43
L53816_LS1 N=91
L5274_LS0 N=87
L5033_LS0 N=88
L4971_LS0 N=199
L43503_LS0 N=19
L4203_LS0 N=663
L4071_LS0 N=116
L37144_LS1 N=116
L3588_LS0 N=309
L35342_LS1 N=8
L34922_LS1 N=24
L3311_LS0 N=61
L31853_LS0 N=123
L3168_LS0 N=67
L3023_LS0 N=7
L29512_LS0 N=37
L148545_LS0 N=47
L14760_LS0 N=266
L13627_LS0 N=176
L136091_LS1 N=147
L12678_LS0 N=75
proportion (%)
Normalized Distance
Distribution
Unrelated individual comparison
Same individual comparison
Threshold
5% FDR
Youden index

Supplementary Figure 4: Temperate phages in MGS metaviromes, gut virome associations with phenotypes and within-individual virus strain variation.

a, Relative abundance of temperate bacteriophages in MGS metaviromes of infants and mothers over time. Linear mixed-effects models (LMM) assessed the relationship between the family member (mother, infant) and the abundance of temperate bacteriophages (Supplementary Data 25). The sample sizes are detailed in Fig. 1a under (total metagenomes section). **b,** Richness variation of temperate bacteriophages in infant MGS metaviromes. Boxplot colours represent the feeding mode. LMM evaluated the relationship between the feeding mode and the richness of temperate bacteriophages in n=30 infants with available feeding mode data over 6 timepoints (n (M1)=26, n(M2)=30, n(M3)=28, n(M6)=27, n(M9)=27, n(M12)=27). **c,** Boxplots showing the difference in clr-transformed abundance of *Akkermansia muciniphila* in infants by birth location (all timepoints are pooled for the figure). **d,** Boxplots showing the difference in clr-transformed aggregated relative abundance of *A. muciniphila* phages in infants by birth location. **e,** Correlation between clr-transformed abundance of *A. muciniphila* and clr-transformed aggregated abundance of its phages. In **c**-**e**, LMMs assessed the relationship between abundances of *A. muciniphila* phages and *A. muciniphila* over time in 32 infants (Fig. 1a, total metagenomes). **f,** Increase in sharedness of infant vOTUs with maternal pre/post-pregnancy vOTUs upon including prophages, analysed using LMM (Supplementary Data 38). **g,** Distribution curves comparing virus genetic distances within individuals (blue) and between unrelated individuals (red) for 28 viruses that showed lower genetic distance in related compared to unrelated mother-infant pairs. X-axis shows genetic distances normalized per virus' median genetic distance. Strain identity thresholds were determined by Youden's index (black dashed line) or empirical FDR (red dashed line, Methods). The N in each histogram corresponds to the number of same-individual comparisons. In **a**-**d**, **f**, each boxplot visualizes the median, hinges (25th and 75th percentiles), and whiskers extending up to 1.5 times the interquartile range from the hinges, representing the spread of the data. Source data are provided as a Source Data file.

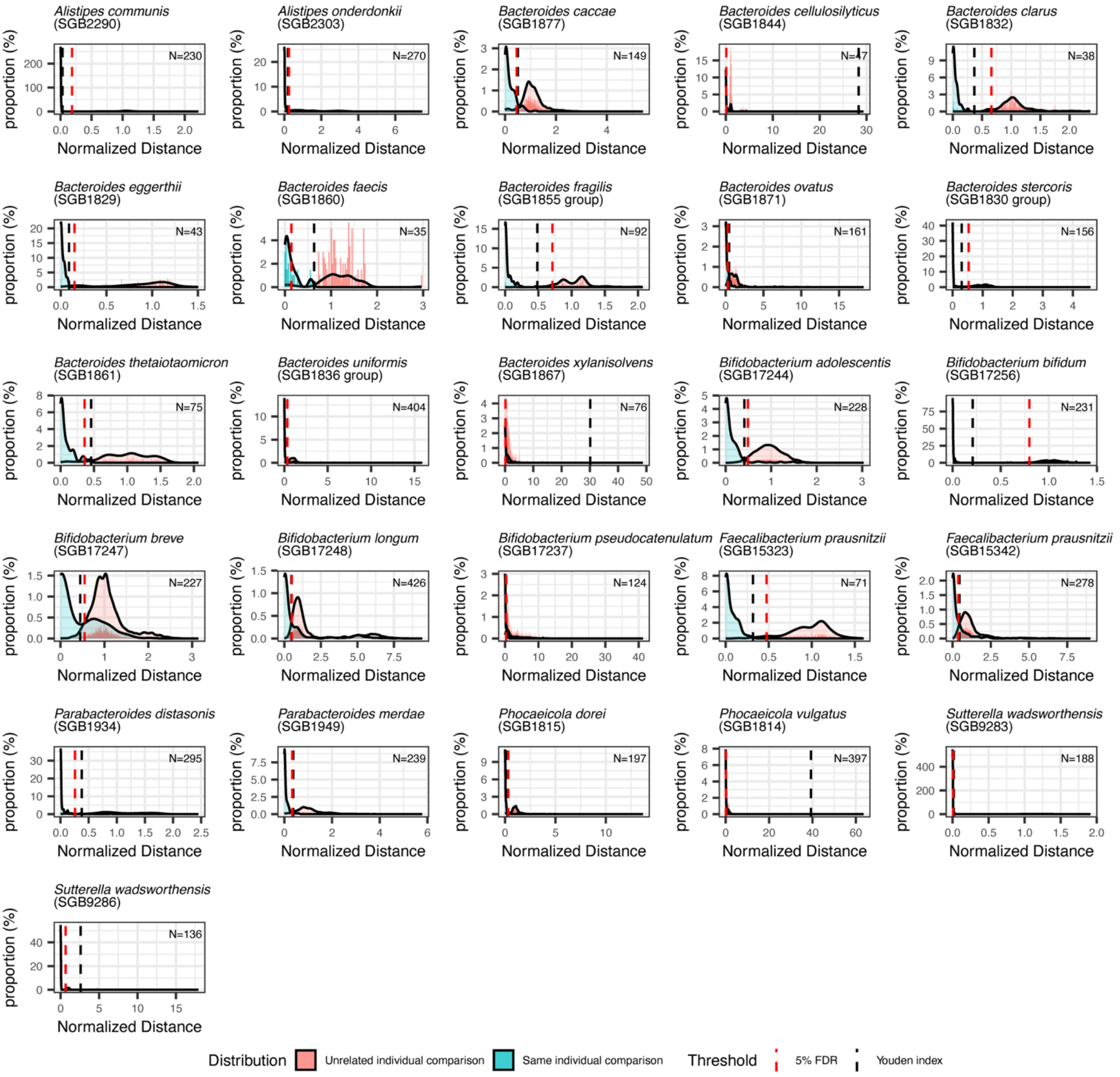

Supplementary Figure 5: Within-individual bacterial strain variation

Distribution curves comparing bacterial strain genetic distances within individuals (blue) and between unrelated individuals (red) for 26 strains predicted to be hosts for transmitted viruses and showing lower genetic distance in related compared to unrelated mother-infant pairs. X-axis shows genetic distances normalized per bacterium' median genetic distance. Strain identity thresholds were determined by Youden's index (black dashed line) or empirical FDR (red dashed line, Methods). The

N in each histogram corresponds to the number of same-individual comparisons. Source data are provided as a Source Data file.

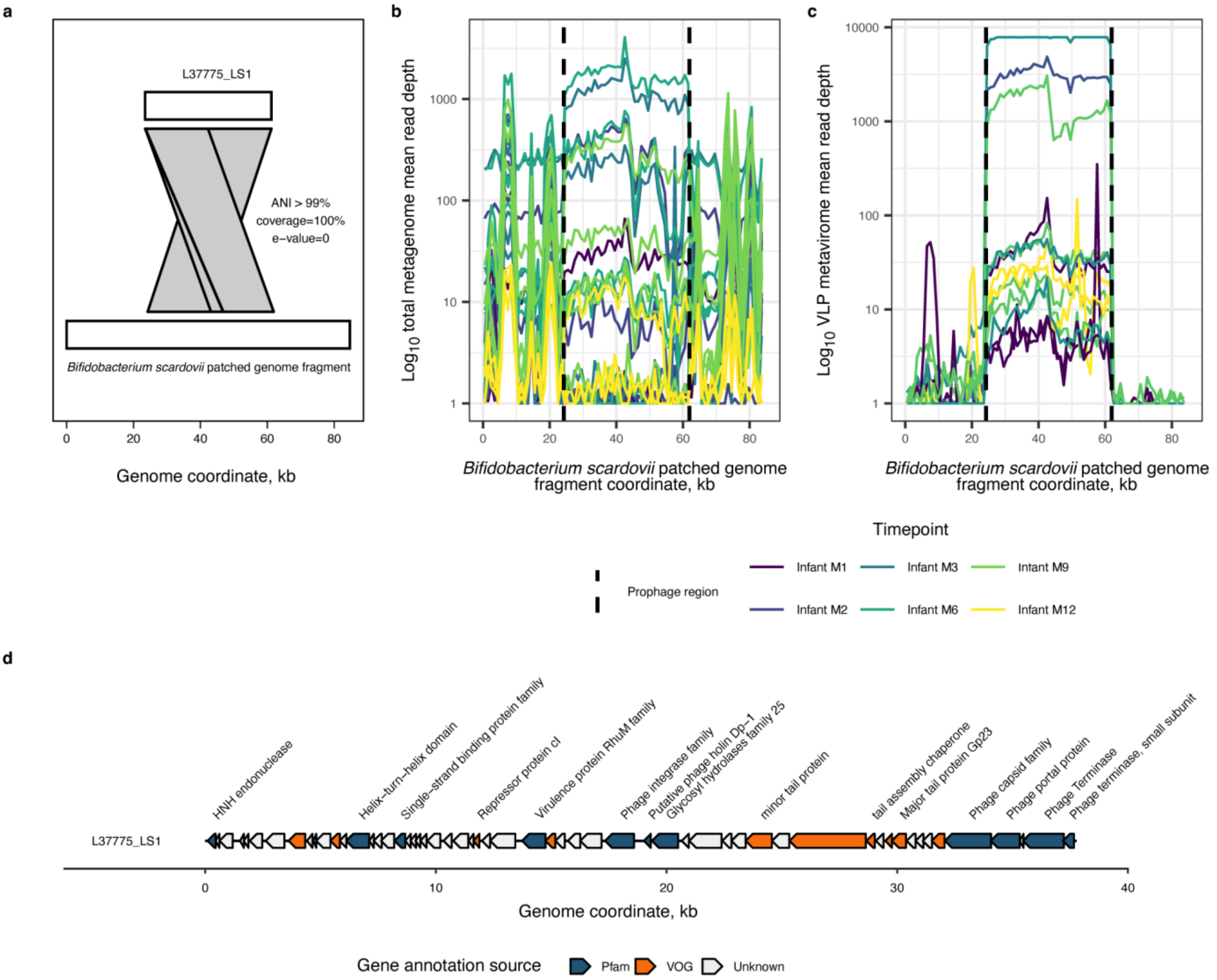

Supplementary Figure 6: Example of virus-host co-transmission not rooted in the maternal gut: a temperate phage of *Bifidobacterium scardovii*

a, Synteny plot of L37775_LS1 genome sequence mapping to the *Bifidobacterium scardovii* patched genome fragment. X-axis indicates the genome coordinates (in kilobases). Lines connecting the L37775_LS1 and *B. scardovii* genome fragment indicate the prophage insertion region. **b**, Depth of the *B. scardovii* patched genome fragment coverage by sequencing reads from total metagenomes positive for *B. scardovii,* and **c**, VLP metaviromes positive for L37775_LS1. Each colour line corresponds to an infant sample and represents mean depth in a 1,001-nt sliding window. Line colours represent samples from different infant timepoints. X-axis depicts the genome coordinate in kilobases. Dashed lines indicate the prophage insertion region. **d,** Genome organization of L37775_LS1. X-axis depicts the genome

coordinates in kilobases. Every predicted protein is represented by a polygon, and its orientation indicates the location of the predicted protein at the positive (right-orientation) or negative (left-orientation) strands. Colours indicate the source of protein annotation: Pfam (blue), VOG (orange). Proteins with no functional annotations are shown in white. Hypothetical proteins of unknown function are shown in respective colours without text annotation.
