# Supplementary material for: Transmission and dynamics of mother-infant gut viruses during pregnancy and early life
Source: Nat Commun. 2024 Mar 2;15:1945. doi: 10.1038/s41467-024-45257-4 (PMC10908809; doi:10.1038/s41467-024-45257-4)
Supplement: Supplementary file 3 — Description of Additional Supplementary Files [file 41467_2024_45257_MOESM3_ESM.pdf]

Description of Additional Supplementary files

Transmission and Dynamics of Mother-Infant Gut Viruses during Pregnancy and Early Life

Garmaeva and Sinha et al.

Supplementary Data 1: Summary statistics of study population.
Supplementary Data 2: Linear mixed-effects model summary for differences in vOTU richness between mothers and infants.
Supplementary Data 3: Linear mixed-effects model summary for NMDS 1 variation over time in infants (virome).
Supplementary Data 4: Linear mixed-effects model summary for NMDS 1 variation over time in infants (bacteriome).
Supplementary Data 5: Linear mixed-effects model summary for NMDS 1 variation between mothers and infants (virome).
Supplementary Data 6: Linear mixed-effects model summary for NMDS 1 variation between mothers and infants (bacteriome).
Supplementary Data 7: Linear mixed-effects model summary for NMDS 1 variation over time in mothers (virome).
Supplementary Data 8: Linear mixed-effects model summary for NMDS 1 variation over time in mothers (bacteriome).
Supplementary Data 9: Linear mixed-effects model summary for viral alpha diversity difference between mothers and infants.
Supplementary Data 10: Linear mixed-effects model summary for bacterial alpha diversity difference between mothers and infants.
Supplementary Data 11: Linear mixed-effects model summary for viral alpha diversity in infants over time.
Supplementary Data 12: Linear mixed-effects model summary for bacterial alpha diversity in infants over time
Supplementary Data 13: Linear mixed-effects model summary for viral alpha diversity in mothers over time.
Supplementary Data 14: Linear mixed-effects model summary for bacterial alpha diversity in mothers over time.
Supplementary Data 15: Linear mixed-effects model summary for retention of various M1 parameters in infants over time.
Supplementary Data 16: Linear mixed-effects model summary for fractions abundance comparisons in infants (virome).

Supplementary Data 17: Linear mixed-effects model summary for fractions sizes comparisons in infants (bacteriome).
Supplementary Data 18: Linear mixed-effects model summary for fractions abundance comparisons in infants (bacteriome).
Supplementary Data 19: Linear mixed-effects model summary for fractions sizes comparisons in mothers (bacteriome).
Supplementary Data 20: Linear mixed-effects model summary for fractions abundance comparisons in mothers (bacteriome).
Supplementary Data 21: Linear mixed-effects model summary for dynamics of highly prevalent vOTU host-based aggregates.
Supplementary Data 22: Linear mixed-effects model summary for dynamics of highly prevalent bacterial genera.
Supplementary Data 23: Linear mixed-effects model summary for relative abundance of active temperative phages difference between mothers and infants.
Supplementary Data 24: Linear mixed-effects model summary for relative abundance of active temperative phages in infants over time.
Supplementary Data 25: Linear mixed-effects model summary for relative abundance of temperate phages in MGS metaviromes in mother versus infant.
Supplementary Data 26: Linear mixed-effects model summary for viral alpha diversity association with infant phenotypes over time.
Supplementary Data 27: Linear mixed-effects model summary for viral alpha diversity associations with infant phenotypes over time corrected for bacterial diversity.
Supplementary Data 28: Linear mixed-effects model summary for associations of active temperate phages richness with phenotypes.
Supplementary Data 29: Linear mixed-effects model summary for associations of temperate phages richness with feeding mode corrected for viral richness.
Supplementary Data 30: Linear mixed-effects model summary for associations of temperate phages richness in MGS metaviromes with feeding mode corrected for bacterial richness.
Supplementary Data 31: Linear mixed-effects model summary for associations of the number of active temperate phages aggregated by host with feeding mode.
Supplementary Data 32: Linear mixed-effects model summary for associations of the number of active temperate phages aggregated by host with feeding mode corrected for host abundance.
Supplementary Data 33: Linear mixed-effects model summary for associations of bacteria with feeding mode.

Supplementary Data 34: Linear mixed-effects model summary for associations of active temperate phages aggregated by host with feeding mode corrected for host abundance and prophages.
Supplementary Data 35: Linear mixed-effects model summary for associations of bacterial species with phenotypes.
Supplementary Data 36: Linear mixed-effects model summary for associations of vOTUs aggregated by host with infant phenotypes.
Supplementary Data 37: Linear mixed-effects model summary for the percentage of shared vOTUs between infants and mothers pre- and post-pregnancy.
Supplementary Data 38: Linear mixed-effects model summary for the percentage of shared vOTUs between infants and mothers pre and post pregnancy adjusted for prophages.
Supplementary Data 39: Viral and Bacterial Strain transmission between mother-infant pairs
Supplementary Data 40: Viral and bacterial co-transmission using Mantel partial correlations.
Supplementary Data 41: Strain co-transmission calculated using the non-random linkage method.
Supplementary Data 42: Metadata for virus genomes and genome fragments reconstructed in the study.
