# Supplementary material for: Transmission and dynamics of mother-infant gut viruses during pregnancy and early life
Source: Nat Commun. 2024 Mar 2;15:1945. doi: 10.1038/s41467-024-45257-4 (PMC10908809; doi:10.1038/s41467-024-45257-4)
Supplement: Supplementary file 5 — Reporting Summary [file 41467_2024_45257_MOESM5_ESM.pdf]

nature portfolio

Corresponding author(s): Alexandra Zhernakova

Last updated by author(s): Dec 18, 2023

Reporting Summary

Nature Portfolio wishes to improve the reproducibility of the work that we publish. This form provides structure for consistency and transparency in reporting. For further information on Nature Portfolio policies, see our Editorial Policies and the Editorial Policy Checklist.

Statistics

For all statistical analyses, confirm that the following items are present in the figure legend, table legend, main text, or Methods section.

n/a	Confirmed	
☐	☒	The exact sample size (*n*) for each experimental group/condition, given as a discrete number and unit of measurement
☐	☒	A statement on whether measurements were taken from distinct samples or whether the same sample was measured repeatedly
☐	☒	The statistical test(s) used AND whether they are one- or two-sided<br>*Only common tests should be described solely by name; describe more complex techniques in the Methods section.*
☐	☒	A description of all covariates tested
☐	☒	A description of any assumptions or corrections, such as tests of normality and adjustment for multiple comparisons
☐	☒	A full description of the statistical parameters including central tendency (e.g. means) or other basic estimates (e.g. regression coefficient) AND variation (e.g. standard deviation) or associated estimates of uncertainty (e.g. confidence intervals)
☐	☒	For null hypothesis testing, the test statistic (e.g. *F*, *t*, *r*) with confidence intervals, effect sizes, degrees of freedom and *P* value noted<br>*Give P values as exact values whenever suitable.*
☒	☐	For Bayesian analysis, information on the choice of priors and Markov chain Monte Carlo settings
☒	☐	For hierarchical and complex designs, identification of the appropriate level for tests and full reporting of outcomes
☐	☒	Estimates of effect sizes (e.g. Cohen's *d*, Pearson's *r*), indicating how they were calculated

Our web collection on statistics for biologists contains articles on many of the points above.

Software and code

Policy information about availability of computer code

Data collection	Data from questionnaires, and birth cards were entered, checked and stored in Utopia Data Management System and was managed by Lifelines by trained data managers.
Data analysis	Open source tools: BBMap (v38.98), KneadData (v0.10.0), FastQC toolkit (v0.11.9), SPAdes (v3.14.1), Prodigal (v2.6.3), HMMER (v3.3.2), VirSorter (v1.0.3), CheckV (v1.0.1), vConTACT2 (v0.11.3), Bowtie2 (v2.4.5), SAMTools (v1.14), iPHoP (v1.2.0), MetaPhlAn4 (Jan 2021 db), kalign (v1.04), StrainPhlAn4, EMBOSS (v6.6.0), minimap2 (v2.25), VIBRANT (v1.2.1)<br>R packages: 'vegan' v2.6-4, 'lmerTest' v3.1-3, 'ape' v5.7-1, 'cutpointr' v1.1.2, 'ggplot2' v.3.4.2, 'ggrepel' v.0.9.3, 'ggforce' v.0.4.1, 'patchwork' v.1.1.2, 'tidyverse' v.2.0.0, 'EnhancedVolcano' v.1.16.0, 'ggforestplot' v.0.1.0, 'corrplot' v.0.92, 'ggtree' v.3.6.2, 'stats' v.4.2.1<br>All codes used in this study can be found at: https://github.com/GRONINGEN-MICROBIOME-CENTRE/LLNEXT_pilot.

For manuscripts utilizing custom algorithms or software that are central to the research but not yet described in published literature, software must be made available to editors and reviewers. We strongly encourage code deposition in a community repository (e.g. GitHub). See the Nature Portfolio guidelines for submitting code & software for further information.

Data

Policy information about availability of data

All manuscripts must include a data availability statement. This statement should provide the following information, where applicable:

- Accession codes, unique identifiers, or web links for publicly available datasets
- A description of any restrictions on data availability
- For clinical datasets or third party data, please ensure that the statement adheres to our policy

Sample information, basic clinical data, family structure, and quality-trimmed and human-contaminant-free sequencing reads can be found in the European Genome-Phenome Archive (EGA) repository (study ID: EGAS00001005969 [https://ega-archive.org/studies/EGAS00001005969]) and available upon submitting a request using the form provided at https://forms.gle/A4Jem2rMnjcygWRD6. A response will be provided within 15 working days. Access to the Lifelines NEXT Project data will be granted to all qualified researchers and will be governed by the provisions laid out in the LLNEXT Data Access Agreement: https://groningenmicrobiome.org/?page_id=2598. This access procedure is in place to ensure that the data is requested solely for research and scientific purposes, in compliance with the informed consent signed by Lifelines NEXT participants. To assist and facilitate data utilization from EGA, authors have prepared a comprehensive instruction guide that can be found at: https://github.com/GRONINGEN-MICROBIOME-CENTRE/LLNEXT_pilot/blob/main/Data_Access_EGA.md. The virus scaffolds and their metadata are available in the Figshare repository under https://doi.org/10.6084/m9.figshare.23926593.v2. Source data are provided with this paper.

Links to other datasets or databases used in our manuscript can be found here:
Human reference genome GRCh38.p13; https://www.ncbi.nlm.nih.gov/datasets/genome/GCF_000001405.39/
MetaPhlAn database of marker genes (mpa_vJan21) ; doi:10.1038/s41587-023-01688-w
ChocoPhlAn SGB database (202103); doi:10.1038/s41587-023-01688-w
GTDB-Tk, database (release207_v2); doi: 10.1093/bioinformatics/btac672
cpn60 database; doi: 10.1101/gr.2649204
COG database (release 2020); doi: 10.1093/nar/gkaa1018
Prokaryotic Virus orthologous Groups (pVOGs); doi: 10.1093/nar/gkw975
NT database (release 249); https://ftp.ncbi.nlm.nih.gov/blast/db/
iPHoP databse (Sept_2021_pub); https://doi.org/10.1371/journal.pbio.3002083
SILVA 138.1 NR99 rRNA genes database; doi: 10.1093/nar/gks1219
ProkaryoticViralRefSeq211-Merged database (release 211); https://ftp.ncbi.nlm.nih.gov/refseq/release/viral/

Research involving human participants, their data, or biological material

Policy information about studies with human participants or human data. See also policy information about sex, gender (identity/presentation), and sexual orientation and race, ethnicity and racism.

Reporting on sex and gender	Our study deals with pregnant women. For their infants we use the term "sex" referring to the biological sex assigned to them by the healthcare professionals at the hospital they were born in.
Reporting on race, ethnicity, or other socially relevant groupings	No socially relevant categorization variables were used in the current manuscript.
Population characteristics	The mother-infant pairs that collected their samples for this study were a part of Lifelines NEXT cohort, a birth cohort designed to study the effects of intrinsic and extrinsic determinants on health and disease in a four-generation design (Warmink-Perdijk 2020, 10.1007/s10654-020-00614-7). Lifelines NEXT is embedded within the Lifelines cohort study, a prospective three-generation population-based cohort study recording the health and health-related aspects of 167,729 individuals living in the Northern Netherlands. From 2016 to 2023, we included 1,450 pregnant Lifelines participants in Lifelines NEXT and intensively followed them, their partners and their children up to at least 1 year after birth. Participant age was collected and used as a covariate for relevant analysis. In addition to this, information regarding gestational age, infant sex, birth weight, feeding mode, mode & place of birth was collected via questionnaires and analyzed in relation to the virome and bacteriome.
Recruitment	This study is part of a larger population cohort study (Lifelines NEXT). Recruitment took place by the healthcare professional of the pregnant woman or through self enrollments of interested participants. All pregnant candidates were invited to participate in Lifelines NEXT without any exclusion criteria. Informed consent forms were available for all participants and all participants were 18 years or older at the time of sample collection. Participants of the present study (n=32) were enrolled from 2016 to 2017, without any bias in selection, besides the availability of longitudinal fecal samples throughout pregnancy and the 1st year of life.
Ethics oversight	The ethical board of the University Medical Centre Groningen approved this study (METc 2015/600). Study procedures were only carried out after a written informed consent was obtained.

Note that full information on the approval of the study protocol must also be provided in the manuscript.

Field-specific reporting

Life sciences study design

All studies must disclose on these points even when the disclosure is negative.

Sample size	No sample size was calculated for our study (32 mother-infants pairs) due to the exploratory nature of the study.
Data exclusions	No data was excluded unless mentioned otherwise. Namely, from 326 successfully sequenced total metagenomes, three low read-depth samples (<5 million reads) and one mislabeled sample were excluded from the analysis. In the vOTU retention analysis, 11 of 32 infants (with VLP metaviromes at M1) and 26 of 30 mothers (with VLP metaviromes at P7) were included. For the bacterial species retention analysis, 28 infants (with M1 samples) and 20 mothers (with P3 samples) were analysed. PPV analysis included 14 infants (with at least 3 timepoints) and 27 mothers (with at least 3 timepoints). All infants (32) and 27 mothers were included in the PPB analysis due to the availability of the required timepoints. In the analyses concerning feeding practices, 28 infants were included due to the availability of VLP metaviromes and feeding data. In the analyses concerning place of delivery, all infants were included
Replication	Results were compared to findings in literature where possible. No replication of the major result of this study (namely the co-transmission of viruses and bacteria from mother to infant) was performed due to the lack of studies with both VLP and metagenomic bacterial data in both mothers and infants.
Randomization	N/A, not an intervention study
Blinding	N/A, not an intervention study

Reporting for specific materials, systems and methods

We require information from authors about some types of materials, experimental systems and methods used in many studies. Here, indicate whether each material, system or method listed is relevant to your study. If you are not sure if a list item applies to your research, read the appropriate section before selecting a response.

Materials & experimental systems

n/a	Involved in the study
☒	☐ Antibodies
☒	☐ Eukaryotic cell lines
☒	☐ Palaeontology and archaeology
☒	☐ Animals and other organisms
☒	☐ Clinical data
☒	☐ Dual use research of concern
☒	☐ Plants

Methods

n/a	Involved in the study
☒	☐ ChIP-seq
☒	☐ Flow cytometry
☒	☐ MRI-based neuroimaging

Plants

Seed stocks	*Report on the source of all seed stocks or other plant material used. If applicable, state the seed stock centre and catalogue number. If plant specimens were collected from the field, describe the collection location, date and sampling procedures.*
Novel plant genotypes	*Describe the methods by which all novel plant genotypes were produced. This includes those generated by transgenic approaches, gene editing, chemical/radiation-based mutagenesis and hybridization. For transgenic lines, describe the transformation method, the number of independent lines analyzed and the generation upon which experiments were performed. For gene-edited lines, describe the editor used, the endogenous sequence targeted for editing, the targeting guide RNA sequence (if applicable) and how the editor was applied.*
Authentication	*Describe any authentication procedures for each seed stock used or novel genotype generated. Describe any experiments used to assess the effect of a mutation and, where applicable, how potential secondary effects (e.g. second site T-DNA insertions, mosiacism, off-target gene editing) were examined.*
